# Measuring distance from the incisors to the esophageal cancer by FDG PET/CT: endoscopy as the reference

**DOI:** 10.1186/s12876-022-02206-z

**Published:** 2022-03-17

**Authors:** Szu-Wei Hsu, Jeffrey S. Chang, Wei-Lun Chang, Forn-Chia Lin, Nan-Tsing Chiu

**Affiliations:** 1 Division of Nuclear Medicine, Department of Medical Imaging, College of Medicine, National Cheng Kung University Hospital, National Cheng Kung University, 138 Sheng-Li Road, Tainan, 704 Taiwan; 2 National Institute of Cancer Research, National Health Research Institutes, 367 Sheng-Li Road, Tainan, 704 Taiwan; 3 Division of Gastroenterology and Hepatology, Department of Internal Medicine, College of Medicine, National Cheng Kung University Hospital, National Cheng Kung University, 138 Sheng-Li Road, Tainan, 704 Taiwan; 4 Division of Radiation Oncology, Department of Oncology, College of Medicine, National Cheng Kung University Hospital, National Cheng Kung University, 138 Sheng-Li Road, Tainan, 704 Taiwan

**Keywords:** 18F-FDG PET/CT, Esophageal neoplasms, Radiotherapy, Endoscopy

## Abstract

**Background:**

Using endoscopy as the reference, this study evaluated the accuracy of 18F-fluorodeoxyglucose positron emission tomography/computed tomography (FDG PET/CT) in measuring distance from the incisors to the PET detectable esophageal cancer. If there is high concordance between endoscopic and PET measurements, our results may provide a basis to use FDG PET/CT in cooperation with endoscopic measurement to localize those PET/CT and CT undetectable esophageal tumors for radiotherapy planning.

**Materials:**

Esophageal cancer patients with pretreatment endoscopy and FDG PET/CT detectable esophageal tumors were recruited retrospectively. The distances from the incisors to the proximal esophageal tumor margins were determined by endoscopy and by the sagittal images of FDG PET/CT. The endoscopic measurement was used as the comparative reference. A nuclear medicine doctor and a radiation oncologist each performed the FDG PET/CT measurement twice for every patient. We analyzed the differences in these measurements, and assessed agreement and reproducibility of the results by the intraclass correlation coefficient (ICC).

**Results:**

Thirty-four patients, with 35 esophageal tumors, were included. By endoscopy and FDG PET/CT, the mean distances from the incisors to the proximal esophageal tumor margin were 27.3 ± 6.4 cm (range 17.1–40.0 cm) and 26.8 ± 6.3 cm (range 15.7–41.3 cm), respectively. The mean absolute differences between the endoscopic and four FDG PET/CT measurements ranged from 1.129 to 1.289 cm (SD: 0.98–1.19). The measurement agreement between FDG PET/CT and endoscopy by ICC was between 0.962 and 0.971. The intra- and interobserver reproducibilities of the two readers were excellent (intraobserver ICC: 0.985, 0.996; interobserver ICC: 0.976–0.984).

**Conclusions:**

FDG PET/CT was in high agreement with endoscopy in measuring the distance from the incisors to the proximal esophageal tumor margin. For FDG PET/CT and CT undetectable esophageal cancer, incorporation of the endoscopic measurement with PET/CT might be a way for making radiotherapy plan.

**Supplementary Information:**

The online version contains supplementary material available at 10.1186/s12876-022-02206-z.

## Background

Esophageal cancer ranks as the seventh most common cancer worldwide [1]. Radiotherapy plays an important role in the treatment of esophageal cancer. 18F-fluorodeoxyglucose positron emission tomography/computed tomography (FDG PET/CT), in addition to providing better staging, monitoring treatment response, and restaging, is also valuable for radiotherapy planning through a more accurate localization of the malignant tumor to improve locoregional disease control and reduce radiation-induced complications [2]. However, early-stage esophageal cancer with small volume may not be detected by FDG PET or CT owing to the limitation of spatial resolution, and it will be difficult to accurately delineate the esophageal tumor on the PET or CT images for radiotherapy planning. In such cases, endoscopic findings would be the only basis for tumor delineation in radiation treatment.

The distance from the incisors to the esophageal tumor margin was recommended to be measured routinely during endoscopy [3]. By mimicking the pathway of the endoscope on the FDG PET/CT sagittal images, the distance from the incisors to an FDG PET/CT visible esophageal cancer margin or an esophageal site is possible to be measured. If the endoscopy and PET/CT measurements in PET visible esophageal tumor are highly concordant, it might be able to use the endoscopic information to locate a PET/CT undetectable esophageal tumor on the PET/CT sagittal images for radiotherapy planning. However, given the endoscopic measurement as the reference, the accuracy of FDG PET/CT measurement remains unknown.

Therefore, we enrolled patients with pretreatment endoscopy and FDG PET/CT visible esophageal cancer. We analyzed the concordance between endoscopy and PET/CT in measuring the distance from the incisors to the proximal esophageal tumor margin.

## Methods

### Patients

We retrospectively reviewed the medical records of all patients who had histopathologically confirmed esophageal cancer and received radiotherapy between January 2015 and September 2020. Patients with pretreatment endoscopy and FDG PET/CT visible esophageal cancer were included in the current study, but those with prior esophageal operation were excluded. The institutional review board of the National Cheng Kung University Hospital approved this retrospective study with a waiver of informed consent.

### Endoscopy

The patients underwent endoscopy examination in left lateral decubitus position by experienced endoscopists with a conventional endoscope (GIF‐H290, Olympus Corp., Tokyo, Japan) before any treatment for esophageal cancer. The distances from the incisors to the proximal and distal esophageal tumor margins were recorded routinely in our hospital, but only the distances of the proximal tumor margins were measured in those endoscope non-traversable tumors.

### FDG PET/CT imaging

For FDG PET/CT imaging, all patients ingested nothing but water for about 6 h. The serum glucose level was checked before the injection of the radiotracer to ensure a level under 200 mg/dl. One hour after intravenous injection of 370 MBq (10 mCi) of FDG, images were acquired by a PET/CT scanner (Biograph mCT flow, Siemens, Germany) as patients were in supine position. A non-contrast-enhanced low-dose CT (120 kVp, CARE Dose, pitch 0.8; reconstructed with a soft tissue kernel, slice thickness 3 mm (for CT images) or 5 mm (for attenuation correction CT), increment 2 mm) was performed first. Subsequently, PET was started in 3-dimmensional mode (matrix 200 × 200, flow motion: 0.7 cm/min (head and neck), 1.2 cm/min (trunk), 2.1 cm/min (legs)). The emission data were corrected for randoms, scatter, and decay, then were reconstructed with an ordered-subset expectation maximization algorithm (2 iterations/21 subsets, with application of point spread function, time-of-flight, and gaussian filtering to a transaxial resolution of 5 mm at full-width at half-maximum). Attenuation correction was performed by using CT data.

### FDG PET/CT analysis

**Fig. 1:**
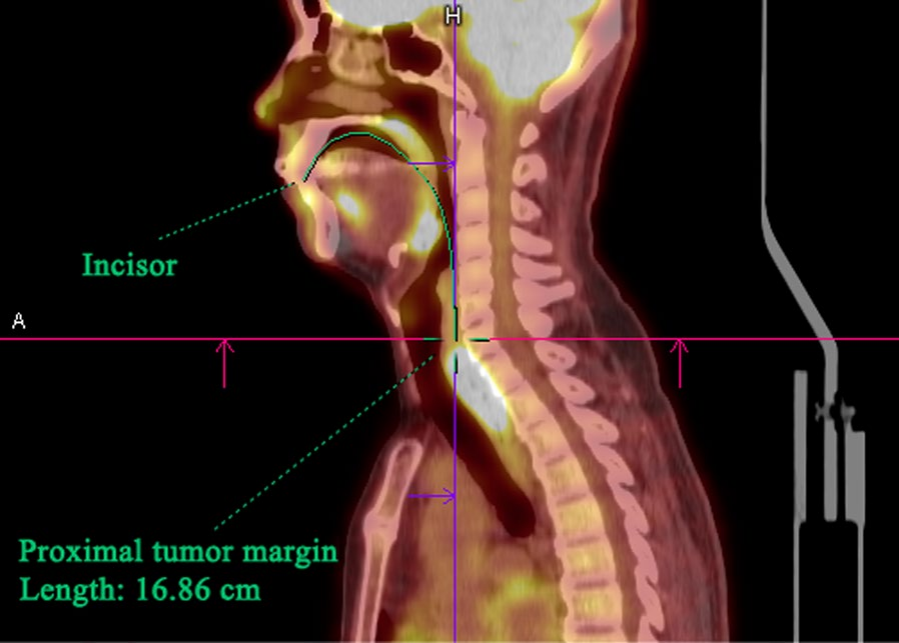
By mimicking the pathway of the endoscope, a polyline (green) was drawn from the patient’s incisor along the oral cavity, inferior margin of the palate, pharynx, and esophagus to the proximal esophageal tumor margin on the fused sagittal PET/CT image

A commercial software (Syngo.via; Siemens Medical Solutions) was used for interpretation and analysis. The attenuation-corrected FDG PET, CT, and PET/CT fusion images in the transaxial, coronal, and sagittal planes as well as maximum intensity projection (MIP) images were displayed. The lower and upper standardized uptake value (SUV) window thresholds of PET images, displayed on a linear grey scale, were set at 0 and 5, respectively. CT images were displayed on soft tissue window. For the PET/CT fusion images, PET images were set in hot-body color scale. The cine MIP was reviewed first to find the esophageal tumor. The most proximal part of the esophageal tumor was identified by reviewing the MIP, transaxial, coronal, and sagittal images, and then a maker was put there on the fused sagittal PET/CT image (Additional file 1: Fig. S1). By mimicking the pathway of the endoscope, a polyline was drawn from the patient’s incisor along the oral cavity, inferior margin of the palate, pharynx, and esophagus to the marker on the fused sagittal PET/CT image, and the length of the line was recorded (Fig. 1). Two physicians, including one nuclear medicine doctor (N.T.C., with 15 years of experience in PET/CT reading) and one radiation oncologist (F.C.L.), blinded to the clinical data of the patients except existence of esophageal cancer made the measurements independently. For each patient, the measurements were performed for two times by each physician with an interval of at least 1 month. The maximal SUV (SUVmax) and mean SUV (SUVmean) of the esophageal tumor were assessed by the nuclear medicine doctor (N.T.C.). A threshold of 40% of the SUVmax within the esophageal tumor was used to delineate the tumor contours for determination of SUVmean.

Before the above measurements, the two doctors practiced the measurement procedures with three pre-treatment esophageal cancer patients’ FDG PET/CT which were performed after October 2020. The readers knew the results of endoscopic measurements and underwent the measurements according to the above procedures.

### Statistical analysis

Descriptive statistics were used to summarize the patient characteristics and differences in the measurements. Data normality was examined by Shapiro–Wilk test. The intra- and inter-observer reproducibility were assessed by the intraclass correlation coefficient (ICC) with a model of absolute agreement and interpreted according to Ko and Li (< 0.5, poor reproducibility; 0.50–0.75, fair reproducibility; 0.75–0.90, good reproducibility; 0.90–1, excellent reproducibility) [4]. ICC and Bland–Altman plot were utilized to examine the agreement between FDG PET/CT and endoscopic measurements. All statistical analyses were performed with SPSS Statistics software (version 17, IBM, NY, USA) and SAS (version 9.4, SAS Institute Inc, NC, USA), and a two-tailed *P* value of < 0.05 was considered significant.

## Results

### Patient characteristics, endoscopic measurements, and FDG PET/CT results

A total of 34 consecutive patients, with 35 esophageal tumors, met our selection criteria. There were 33 men and one woman (age range, 40–76 years; mean age ± standard deviation, 57.4 ± 8.0 years). The cases were all squamous cell carcinoma except for one case of spindle cell carcinoma. Thirty-two patients underwent concurrent chemoradiotherapy, one patient received concurrent chemoradiotherapy with subsequent operation, and one patient had radiotherapy alone. There was no significant deviation or tortuosity of uninvolved esophagus proximal to the tumor in our patients.

The interval between FDG PET/CT and endoscopy was 14.2 ± 7.9 days (range 2–35 days). The mean distance from the incisors to the proximal esophageal tumor margin was 27.3 ± 6.4 cm (range 17.1–40.0 cm) by endoscopy and was 26.8 ± 6.3 cm (range 15.7–41.3 cm) by FDG PET/CT. The mean SUVmax was 17.7 ± 11.2 (range 3.52–68.93), and the mean SUVmean was 10.6 ± 6.7 (range 2.07–40.5). Table 1 shows the summary of patient characteristics, endoscopic measurements, and FDG PET/CT results.

**Table 1:** Summary of patient characteristics (n = 34), results of FDG PET/CT, and endoscopic measurements

Variable	Value
Age (years)^a^	57.4 (8.0; 40–76)
Gender	
Male	33
Female	1
T category	
1	2
2	3
3	28
4	1
N category	
1	8
2	10
3	16
Mean distance from incisors to proximal tumor margin by endoscopy (cm)^a^	27.3 (6.4; 17.1–40.0)
Mean distance from incisors to proximal tumor margin by FDG PET/CT (cm)^a^	26.8 (6.3; 15.7–41.3)
Mean SUVmax^a^	17.7 (11.2; 3.52–68.93)
Mean SUVmean^a^	10.6 (6.7; 2.07–40.5)

*SUVmax* maximal SUV of esophageal tumor, *SUVmean* mean SUV of esophageal tumor

^a^Numbers in parentheses are standard deviation and ranges

### Agreement between endoscopic and FDG PET/CT measurements

**Fig. 2:**
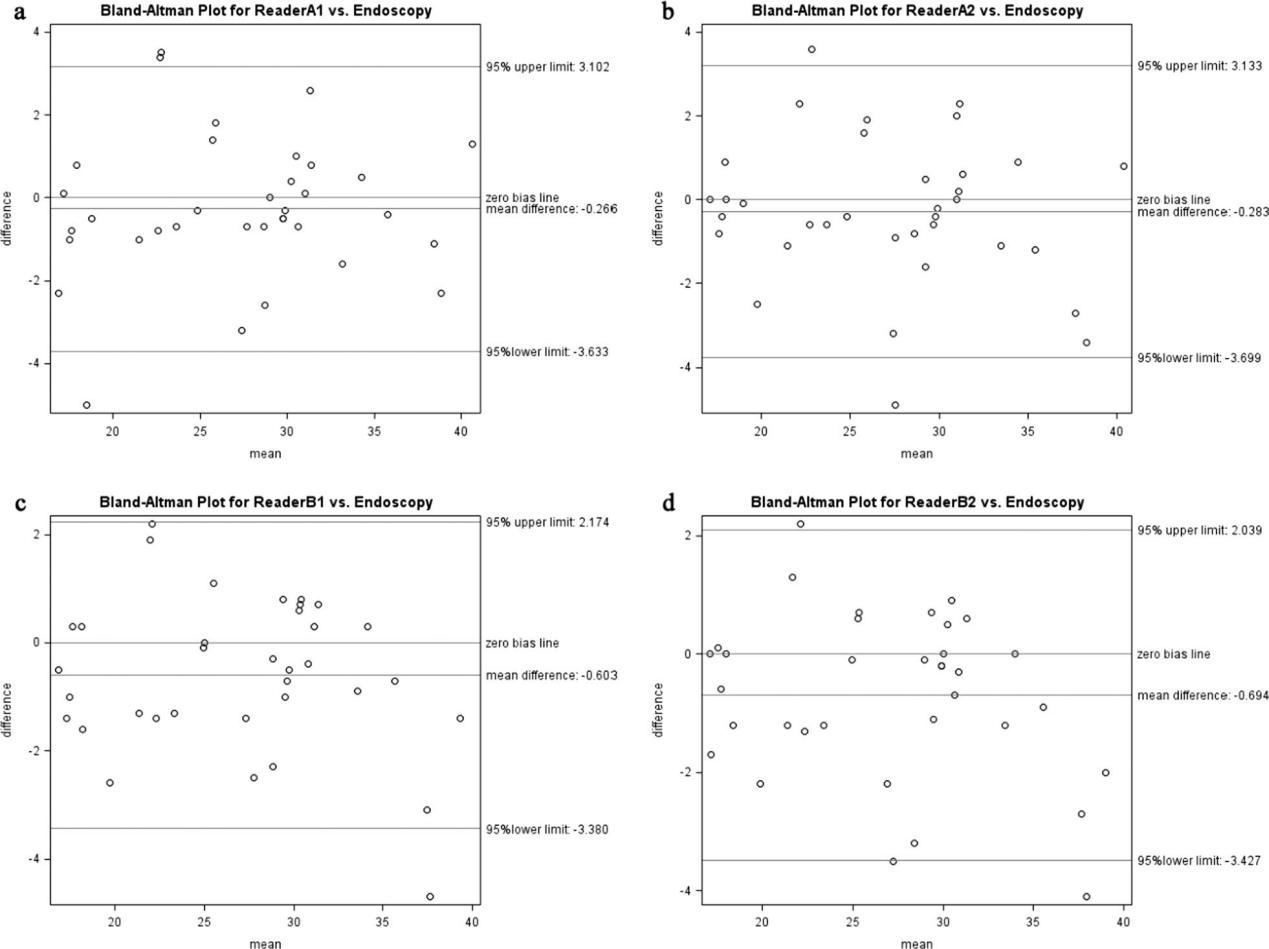
Bland–Altman plot for FDG PET/CT versus endoscopy. **a** Reader A exam1 versus endoscopy. **b** Reader A exam2 versus endoscopy. **c** Reader B exam1 versus endoscopy. **d** Reader B exam2 versus endoscopy. *Reader A* radiation oncologist, *Reader B* nuclear medicine doctor

The mean absolute differences between the endoscopic and four FDG PET/CT measurements ranged from 1.129 to 1.289 cm (SD: 0.98–1.19, Table 2). ICC showed comparable measurement between FDG PET/CT and endoscopic measurements (ICC: 0.962–0.971, Table 2). Bland–Altman plot analysis indicated that the 95% limits of localization difference (FDG PET/CT measurement minus endoscopic measurement) between the endoscopic and the two repeated FDG PET/CT measurements by each reader were − 3.633 to 3.102 cm and − 3.699 to 3.133 cm for reader A (radiation oncologist), and − 3.380 to 2.174 cm and − 3.427 to 2.039 cm for reader B (nuclear medicine doctor) (Fig. 2). Table 3 showed the probabilities of measurement differences within 1 cm, 2 cm, and 3 cm between the endoscopic and FDG PET/CT measurements.

**Table 2:** Reproducibility of FDG-PET measurements and agreement between endoscopic and FDG PET/CT measurements

Comparison group	Mean absolute difference	ICC
Reader A exam1 versus endoscopy^a^	1.277 cm (1.16; 0.00–5.00)	0.965
Reader A exam2 versus endoscopy^a^	1.289 cm (1.19; 0.00–4.90)	0.962
Reader B exam1 versus endoscopy^a^	1.174 cm (0.98; 0.00–4.70)	0.971
Reader B exam2 versus endoscopy^a^	1.129 cm (1.06; 0.00–4.10)	0.970
Reader A exam1 versus Reader A exam2^a^	0.663 cm (0.90; 0.00–4.60)	0.985
Reader B exam1 versus Reader B exam2^a^	0.497 cm (0.30; 0.00–1.00)	0.996
Reader A exam1 versus Reader B exam1^a^	0.897 cm (0.72; 0.00–2.70)	0.984
Reader A exam2 versus Reader B exam2^a^	0.954 cm (0.97; 0.00–4.90)	0.976
Reader A exam1 versus Reader B exam2^a^	0.994 cm (0.80; 0.00–3.30)	0.980
Reader A exam2 versus Reader B exam1^a^	0.834 cm (0.77; 0.10–4.60)	0.983

*Reader A* radiation oncologist, *Reader B* nuclear medicine doctor, *ICC* intraclass correlation coefficient

^a^Numbers in parentheses are standard deviation and ranges

**Table 3:** Possibilities of measurement differences within 1 cm, 2 cm, and 3 cm between endoscopic and FDG PET/CT measurements

Comparison group	% within 1 cm (95% CI)	% within 2 cm (95% CI)	% within 3 cm (95% CI)
Reader A exam1 versus endoscopy	62.9% (46.9–78.9%)	77.1% (63.2–91.1%)	88.6% (78.0–99.1%)
Reader A exam2 versus endoscopy	57.1% (40.8–73.5%)	77.1% (63.2–91.1%)	88.6% (78.0–99.1%)
Reader B exam1 versus endoscopy	57.1% (40.8–73.5%)	82.9% (70.4–95.3%)	94.3% (86.6–100.0%)
Reader B exam2 versus endoscopy	54.3% (37.8–70.8%)	80.0% (66.8–93.3%)	91.4% (82.1–100.0%)

*Reader A* radiation oncologist, *Reader B* nuclear medicine doctor, *CI* confidence interval

### Reproducibility of FDG-PET measurements

Analysis of the intra- and inter-observer reproducibility of the two readers showed excellent reproducibility (intraobserver: ICC: 0.985, 0.996; interobserver: ICC: 0.976–0.984; Table 2). The mean absolute differences between the measurements ranged from 0.497 to 0.994 cm (intraobserver: 0.497 cm, 0.663 cm; interobserver: 0.834–0.994 cm; Table 2).

## Discussion

CT simulation and conformal treatment planning are currently recommended in radiotherapy for esophageal cancer [5]. FDG PET/CT has been regarded valuable to provide information for better delineation of the treatment targets [2]. However, if the esophageal tumor is not visible on FDG PET/CT or CT, it can be difficult to determine the radiotherapy field. There exists an unmet medical need for delineation of FDG PET/CT or CT undetectable esophageal tumors in radiotherapy planning.

This study, which included the patients with esophageal cancer detectable on FDG PET/CT, showed that the distance from the incisors to the proximal esophageal tumor margin assessed by FDG PET/CT was highly concordant with the endoscopic measurement. The intra- and interobserver (nuclear medicine doctor, radiation oncologist) agreement was excellent. Applying these results in esophageal cancer not detectable on FDG PET/CT, we can localize the gross tumor volume on FDG PET/CT image by using endoscopic measurement. Accordingly, the clinical and planning target volumes could be sequentially created by adding margins from gross tumor volume and used in radiotherapy [5]. But on the opposite side, discrepancy between endoscopy and FDG PET/CT measurements would exist in cases with deviated or tortuous esophagus. In the present cohort, none of patients had significant esophageal deviation or tortuosity. Further studies are warranted. In addition, discrepancy also possibly derived in part from the different postures between the two examinations. The potential difference between the two measurements should be taken into consideration in the delineation of gross tumor volume.

Accurate definition of the primary esophageal cancer is very important for a successful radiotherapy planning. CT planning is currently the standard method for tumor volume delineation in radiotherapy, but longitudinal boundaries of esophageal tumor may not be clear due to poor soft tissue contrast, and small tumors (T1 or T2) are often not observable on CT [6]. Endoscopy provides precise evaluation of longitudinal tumor boundaries and the distance from incisors to tumor margin was suggested to be measured routinely [3]. The endoscopic measurement has been used to correlate with anatomical landmark on CT images for a better tumor volume delineation. The carina, which frequently located at 25 cm from the incisor teeth, is a commonly adopted anatomical landmark [7]. However, a study showed considerable variability of the carina-incisor distance (CID, mean CID: 25.7 cm, range 20.5–29 cm, SD ± 1.99), and the macroscopic disease would not be properly covered by radiation therapy in 18% of patients if the location of the carina was set at 25 cm from the incisor teeth [8]. To define esophageal tumor margins more accurately, several methods were proposed, including placement of fiducial markers at the esophageal tumor margins [9, 10], or injection of contrast solution into the tumor [11] during endoscopy. Invasiveness, time-consuming, possible migration of fiducial markers, tissue deformation [10, 12], and dissipation of contrast solution [13] are the disadvantages of the above methods, making them not feasible for clinical practice [12, 13]. Using endoscopic ultrasound to record the superior extent of the aortic arch as a reference point and incorporate this information into the CT planning was reported to improve tumor localization [14], but this is not a recommended routine procedure, and besides, the different patient positions during endoscopy and radiotherapy may alter the location of mediastinal structures [13].

For initial workup of newly diagnosed esophageal cancer, the NCCN Clinical Practice Guidelines in Oncology recommend the use of FDG PET/CT if no evidence of M1 disease [5]. Studies supported the application of FDG PET for better determination of gross tumor volume. Good correlation was found in the measurement of esophageal tumor length between FDG PET and surgical pathology results [15, 16], while CT scan overestimated tumor length and may lead to inappropriate radiotherapy planning [17]. Because FDG PET can provide precise localization of esophageal tumor, improvement in radiotherapy planning was demonstrated [18–20]. Nevertheless, FDG PET might not detect small esophageal cancers. Among T1 tumors, the detection rates of 43% [21], 55% [22], 71% [23], and 83% [24] have been reported. This problem may become more common when routine endoscopy screening is recommended in patients with head and neck cancer to detect the synchronous or metachronous esophageal cancer. Routine esophageal screening in head and neck cancer patients showed that the prevalence of second primary esophageal cancer was 4.5% and about 41% of these patients had an early T stage tumor [25]. Our study revealed that the distance from incisors to esophageal tumor margin measured by FDG PET/CT correlated well with the endoscopic measurement. Therefore, translation of the endoscopic information into those FDG PET/CT invisible esophageal tumor for radiotherapy planning is feasible.

The study has several limitations. First, we adopted a fixed SUV window thresholds for PET images reading to reduce observer variability. The upper SUV window threshold was set at 5 because it was about double the SUVmean value of the liver, which was suggested for PET reading [26], in our PET/CT scanner. According to our experience, pathologic and physiological FDG uptake can be reasonably illustrated by this setting. However, alteration of SUV window thresholds may render different FDG PET measurement results, and other PET/CT scanner may have dissimilar optimal SUV window setting. Second, almost all of the esophageal cancers in this study were squamous cell carcinoma and there was no adenocarcinoma. Thus, the results of the current study may not be generalizable to esophageal adenocarcinoma and more studies are warranted. Third, this is a retrospective study. A prospective study to enroll esophageal cancer patients who have non-visible tumor on pretreatment FDG PET/CT and undergo additional endoscopy to place fiducial markers at the esophageal tumor margins is needed to verify the performance of FDG PET/CT measurement. Fourth, the results were from a single center with limited numbers of patients. Different PET/CT scanners and software may have different correlation with endoscopic measurement. A multicenter study with more patients to confirm the current results is necessary.

## Conclusions

Our study indicates that determination of the distance from the incisors to the esophageal tumor margin by FDG PET/CT is comparable with endoscopic measurement and has excellent reproducibility. Our results can be used as a reference to make radiotherapy planning based on the endoscopic measurement and FDG PET/CT in esophageal cancer patients with PET/CT undetectable tumors.

## Supplementary Information

**Additional file 1.** Method to identify the proximal esophageal tumor margin.

## Data Availability

All data generated or analyzed during this study are included in this published article. The datasets used and/or analyzed during the current study are available from the corresponding authors on reasonable request.

## Abbreviations

CID: Carina-incisor distance
CT: Computed tomography
FDG: 18F-fluorodeoxyglucose
ICC: Intraclass correlation coefficient
MIP: Maximum intensity projection
PET: Positron emission tomography
SUV: Standardized Uptake Value
SUVmax: Maximal SUV
SUVmean: Mean SUV

## References

[1] Bray F, Ferlay J, Soerjomataram I, Siegel RL, Torre LA, Jemal A (2018). Global cancer statistics 2018: GLOBOCAN estimates of incidence and mortality worldwide for 36 cancers in 185 countries. CA Cancer J Clin.

[2] Kwee RM, Marcus C, Sheikhbahaei S, Subramaniam RM (2015). PET with fluorodeoxyglucose F 18/computed tomography in the clinical management and patient outcomes of esophageal cancer. Pet Clin.

[3] Varghese TK, Hofstetter WL, Rizk NP (2013). The society of thoracic surgeons guidelines on the diagnosis and staging of patients with esophageal cancer. Ann Thorac Surg.

[4] Koo TK, Li MY (2016). A guideline of selecting and reporting intraclass correlation coefficients for reliability research. J Chiropr Med.

[5] Ajani JA, D'Amico TA, Bentrem DJ, et al. NCCN clinical practice guidelines in oncology: esophageal and esophagogastric junction cancer. Version 4.2020. www.nccn.org.

[6] Torok JA, Perez BA, Czito BG, Wc G, Yin FF, Palta M, Khan FM, Gibbons JP, Sperduto PW (2016). Cancers of the gastrointestinal tract. Khan's treatment planning in radiation oncology.

[7] Czito BG, Palta M, Willett CG, Halperin EC, Brady LW, Wazer DE, Perez CA (2019). Esophageal cancer. Perez and Brady's principles and practice of radiation oncology.

[8] Rice PF, Crosby TL, Roberts SA (2003). Variability of the carina-incisor distance as assessed by endoscopic ultrasound. Clin Oncol (R Coll Radiol).

[9] Pfau PR, Pham H, Ellis R, Das A, Isenberg G, Chak A (2005). A novel use of endoscopic clips in the treatment planning for radiation therapy (XRT) of esophageal cancer. J Clin Gastroenterol.

[10] Machiels M, van Hooft J, Jin P (2015). Endoscopy/EUS-guided fiducial marker placement in patients with esophageal cancer: a comparative analysis of 3 types of markers. Gastrointest Endosc.

[11] Burmeister BH, Beukema J, Guidi R, Harvey JA, Gotley D, Smithers BM (2001). Localization of small esophageal cancers for radiation planning using endoscopic contrast injection: report on a series of eight cases. Dis Esophagus.

[12] Jin P, van der Horst A, de Jong R (2015). Marker-based quantification of interfractional tumor position variation and the use of markers for setup verification in radiation therapy for esophageal cancer. Radiother Oncol.

[13] Leong T, Paulino AC (2008). Esophageal cancer. PET-CT in radiotherapy treatment planning.

[14] Thomas E, Crellin A, Harris K, Swift S, Montefiore DS (2004). The role of endoscopic ultrasound (EUS) in planning radiotherapy target volumes for oesophageal cancer. Radiother Oncol.

[15] Mamede M, El Fakhri G, Abreu-e-Lima P, Gandler W, Nose V, Gerbaudo VH (2007). Pre-operative estimation of esophageal tumor metabolic length in FDG-PET images with surgical pathology confirmation. Ann Nucl Med.

[16] Zhong X, Yu J, Zhang B (2009). Using 18F-fluorodeoxyglucose positron emission tomography to estimate the length of gross tumor in patients with squamous cell carcinoma of the esophagus. Int J Radiat Oncol Biol Phys.

[17] Sillah K, Williams LR, Laasch HU (2010). Computed tomography overestimation of esophageal tumor length: implications for radiotherapy planning. World J Gastrointest Oncol.

[18] Konski A, Doss M, Milestone B (2005). The integration of 18-fluoro-deoxy-glucose positron emission tomography and endoscopic ultrasound in the treatment-planning process for esophageal carcinoma. Int J Radiat Oncol Biol Phys.

[19] Leong T, Everitt C, Yuen K (2006). A prospective study to evaluate the impact of FDG-PET on CT-based radiotherapy treatment planning for oesophageal cancer. Radiother Oncol.

[20] Muijs CT, Schreurs LM, Busz DM (2009). Consequences of additional use of PET information for target volume delineation and radiotherapy dose distribution for esophageal cancer. Radiother Oncol.

[21] Kato H, Miyazaki T, Nakajima M (2005). The incremental effect of positron emission tomography on diagnostic accuracy in the initial staging of esophageal carcinoma. Cancer.

[22] Little SG, Rice TW, Bybel B (2007). Is FDG-PET indicated for superficial esophageal cancer?. Eur J Cardiothorac Surg.

[23] Manabe O, Hattori N, Hirata K (2013). Diagnostic accuracy of lymph node metastasis depends on metabolic activity of the primary lesion in thoracic squamous esophageal cancer. J Nucl Med.

[24] Huang YC, Lu HI, Huang SC (2017). FDG PET using SUVmax for preoperative T-staging of esophageal squamous cell carcinoma with and without neoadjuvant chemoradiotherapy. BMC Med Imaging.

[25] Su YY, Chen WC, Chuang HC (2013). Effect of routine esophageal screening in patients with head and neck cancer. JAMA Otolaryngol Head Neck Surg.

[26] Rogasch JM, Apostolova I, Steffen IG (2016). Standardized visual reading of F18-FDG-PET in patients with non-small cell lung cancer scheduled for preoperative thoracic lymph node staging. Eur J Radiol.

