# Supplementary material for: Measuring distance from the incisors to the esophageal cancer by FDG PET/CT: endoscopy as the reference
Source: BMC Gastroenterol. 2022 Mar 17;22:126. doi: 10.1186/s12876-022-02206-z (PMC8928607; doi:10.1186/s12876-022-02206-z)
Supplement: Supplementary file 1 — Additional file 1. Method to identify the proximal esophageal tumor margin. [file 12876_2022_2206_MOESM1_ESM.docx]

**Figure and legends**


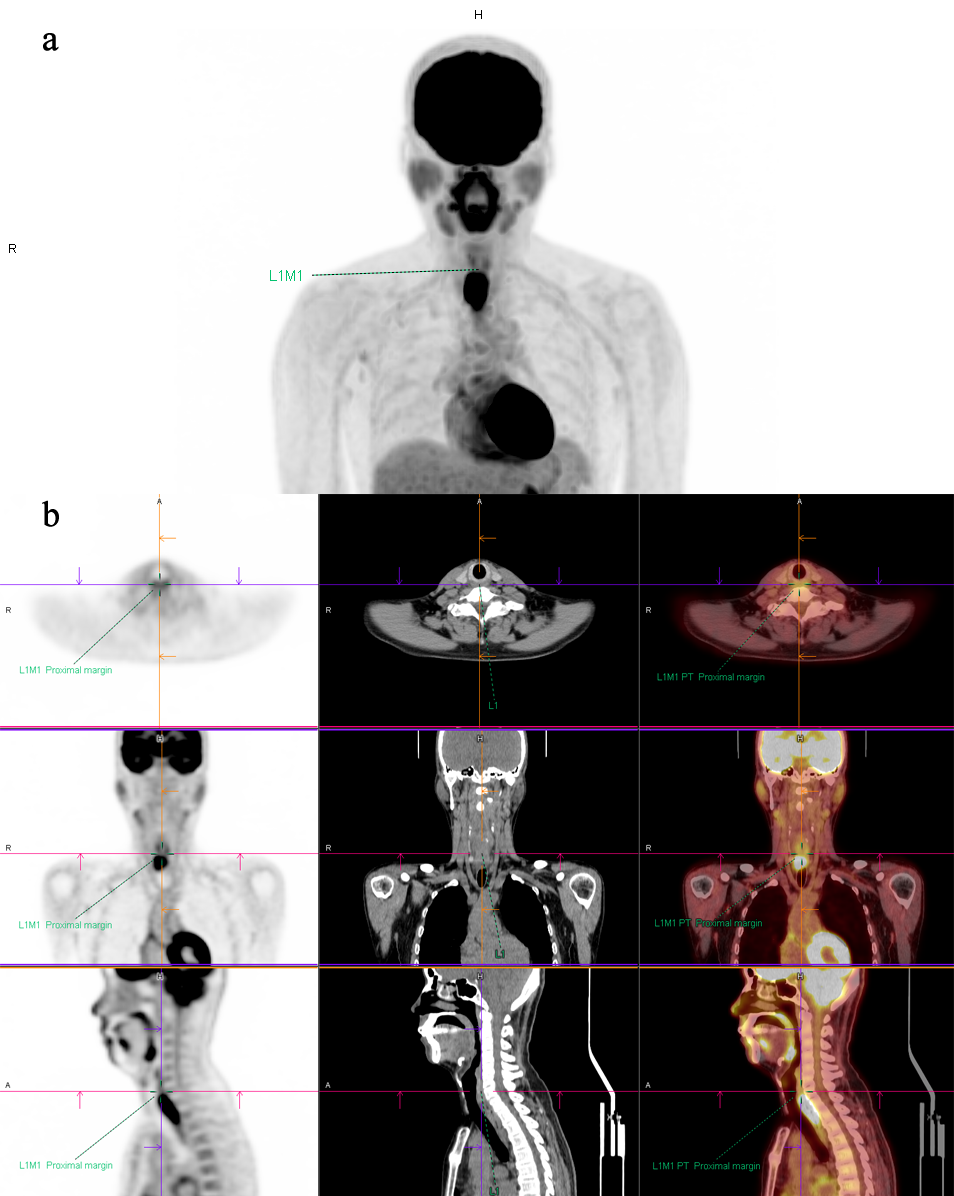


**Additional file 1: Fig. S1.** The proximal esophageal tumor margin was identified by reviewing the MIP (a), transaxial, coronal, and sagittal images (b), and then a maker was put there on the fused sagittal PET/CT image.
